# Circulating Thrombospondin-1 and Endothelin-1 Levels Tend to Decline with Increasing Obesity Severity in Women: Evidence from a Pilot, Cross-Sectional Study

**DOI:** 10.3390/jcm14072143

**Published:** 2025-03-21

**Authors:** Marta Greco, Maria Mirabelli, Luciana Sicilia, Francesco Dragone, Stefania Giuliano, Francesco S. Brunetti, Rosa Scalise, Eusebio Chiefari, Aikaterini Andreadi, Davide Lauro, Daniela P. Foti, Antonio Brunetti

**Affiliations:** 1 Department of Health Sciences, University “Magna Græcia” of Catanzaro, 88100 Catanzaro, Italy; marta.greco@unicz.it (M.G.);; 2 Operative Unit of Clinical Pathology, “R. Dulbecco” Hospital, 88100 Catanzaro, Italy; 3 Operative Unit of Endocrinology, “R. Dulbecco” University Hospital, 88100 Catanzaro, Italy; 4 Section of Endocrinology and Metabolic Diseases, Department of Systems Medicine, University of Rome Tor Vergata, 00133 Rome, Italyd.lauro@med.uniroma2.it (D.L.); 5 Department of Experimental and Clinical Medicine, University “Magna Græcia” of Catanzaro, 88100 Catanzaro, Italy

**Keywords:** obesity, biomarkers, insulin resistance, inflammation, cardiometabolic risk, thrombospondin-1, endothelin-1

## Abstract

**Background:** Thrombospondin-1 (TSP1) is a multimeric glycoprotein that is increasingly recognized as a mediator of metabolic, thrombotic, and inflammatory processes. Although TSP1 expression has been associated with adipose tissue dysfunction and insulin resistance, the precise relationship with obesity severity remains unclear. Endothelin-1 (ET1), another important regulator of vascular homeostasis, may also contribute to obesity-related cardiometabolic risk, with evidence suggesting sex-specific differences, including delayed onset in women. The study aimed to investigate circulating TSP1 and ET1 levels in a cohort of nondiabetic obese female adults, evaluate their associations with metabolic and inflammatory parameters, and determine whether these markers differ according to obesity severity and related disease risk. **Methods:** Fifty-five nondiabetic women with obesity and no history of cardiovascular events were enrolled at the Endocrinology Unit (“R. Dulbecco” Univ. Hospital, Catanzaro, Italy). Anthropometric and clinical data, together with hematological and coagulation parameters and metabolic indices (HOMA-IR, HbA1c, and lipid profile), were evaluated. TSP1 and ET1 concentrations were measured using automated enzyme immunoassays (ELISAs). The participants were stratified by BMI (30–34.9 vs. ≥35 kg/m^2^) into low-risk and moderate/high-risk obesity based on the WHO classification, and correlations between biomarkers and metabolic/inflammatory parameters were evaluated. **Results:** The median BMI was 33.7 kg/m^2^, with 52% of participants having moderate/high-risk obesity (WHO Class II/III). A significant proportion (69.8%) showed insulin resistance (HOMA-IR > 2.5). TSP1 was positively correlated with white blood cell count (WBC, r = 0.354, *p* < 0.01), platelet count (PLT, r = 0.411, *p* < 0.01), and glycated hemoglobin (r = 0.391, *p* < 0.01), suggesting an association with both inflammation and glycemic control. ET1 was positively correlated with liver enzymes and triglycerides but negatively correlated with PLT and D-dimer. Women with moderate/high-risk obesity had significantly higher HOMA-IR, D-dimer, and inflammatory markers, in addition to a lower TSP1-to-PLT ratio. **Conclusions:** In this pilot study, TSP1 and ET1 levels tended to decrease with increasing obesity severity in women but were associated with distinct metabolic and inflammatory profiles. The results support the potential role of TSP1 as a biomarker for obesity-related cardiometabolic risk and highlight the complex interplay between TSP1, ET1, and obesity progression. Further studies may clarify whether targeting TSP1 can ameliorate chronic inflammation and insulin resistance in obesity and the potential sex-specific influences on these mechanisms.

## 1. Introduction

Thrombospondin-1 (TSP1) is a large, multimeric glycoprotein belonging to the thrombospondin family, initially characterized by Baenziger et al. in 1971 [1]. Structurally, TSP1 forms a trimer (~450 kDa) composed of an N-terminal domain, three type I properdin repeats, three type II epidermal growth factor (EGF)-like repeats, a type III calcium-binding region, and a C-terminal domain involved in cell attachment [2]. While TSP1 was originally identified in platelet α-granules for its critical function in platelet aggregation, subsequent studies have revealed its highly regulated expression in diverse cell types, where it participates in multiple biological processes [3].

Although TSP1 plays a canonical role in hemostasis and thrombosis—partly via cAMP-mediated platelet activation [4]—recent data implicate it in metabolic disorders such as obesity, diabetes, and cardiovascular diseases [5]. Specifically, TSP1 has been shown to drive adipose tissue inflammation, affecting macrophage chemotaxis and cytokine release and the angiogenic requirements of expanding adipose depots [6]. Mouse models deficient in TSP1 exhibit resistance to high-fat diet-induced obesity [7], whereas in vitro data indicate that TSP1 expression is downregulated by hypoxia in 3T3-L1 adipocytes, suggesting its potential involvement in obesity-related adipose dysfunction [8].

From an angiogenic perspective, TSP1 may either stimulate or restrain vascular remodeling, depending on the biological context. Its interaction with proteins, such as Argonaute 1 (AGO1), has been linked to the modulation of adipose vascularization and metabolic homeostasis. TSP1 alterations have also been documented in endothelial cells from obese or diabetic donors, implying that TSP1 may reinforce insulin resistance by limiting pro-angiogenic mechanisms [9]. In addition, TSP1 influences inflammatory signaling, in part through the activation of the Toll-like receptor 4 (TLR4) pathway, which enhances the production of pro-inflammatory cytokines (i.e., TNF-α). Clinical studies have reported elevated TSP1 levels among individuals with visceral obesity and related abnormalities (i.e., hypertension and hyperglycemia) [10]. Although increased serum TSP1 may be partially attributable to platelet activation, proteomic analyses of peripheral blood mononuclear cells and adipose tissue consistently implicate TSP1 in obesity and related metabolic dysregulations [11].

The role of TSP1 in cardiovascular disease is substantial, especially in the context of cardiac fibrosis—where it activates matrix metalloproteinases (MMPs) and latent TGFβ1 [12]—along with promoting monocyte/macrophage infiltration and smooth muscle cell proliferation in injured arterial walls [13]. Through its interactions with CD47 and CD36, TSP1 can inhibit nitric oxide (NO)-mediated vasodilation and alter fatty acid uptake, thereby exacerbating endothelial dysfunction and thrombosis [5]. Polymorphisms in the *TSP1* gene have also been tied to greater genetic susceptibility to myocardial infarction, and TSP1 deficiency leads to intensified inflammation following infarction in murine models [14].

Overall, TSP1 has emerged as a multifaceted regulator of metabolic, inflammatory, and thrombotic pathways, offering promise as a therapeutic target for obesity-associated conditions. Building on in vitro observations of reduced TSP1 under hypoxic conditions—reflecting a potential mechanism in obese adipose tissue dysfunction [8]—we conducted a cross-sectional study to examine circulating TSP1 in obese women with varying degrees of obesity severity and related disease risk. We additionally evaluated endothelin-1 (ET1), another protein implicated in metabolic and vascular pathophysiology, to further elucidate cardiometabolic risk in this population.

The interplay between TSP1 and ET1 is primarily mediated through their effects on vascular function and inflammation. ET1 promotes vascular smooth muscle cell (VSMC) contraction by activating ET1 receptors, and TSP1 suppresses endothelial nitric oxide synthase (eNOS) activity, reducing NO bioavailability and enhancing ET1-induced vasoconstriction. Furthermore, TSP1 interacts with CD47 to downregulate c-Myc, a transcription factor that typically inhibits ET1 and ET1 receptor A expression, thus indirectly amplifying ET1 signaling and contributing to vascular dysfunction [15,16]. Studies in CD47-deficient mice have demonstrated lower levels of ET1 receptor A, suggesting that the TSP1/CD47 axis plays a role in ET1-mediated vascular tone regulation [17]. Collectively, these interactions contribute to endothelial dysfunction, increasing the risk of systemic cardiovascular complications, such as hypertension and atherosclerosis.

Despite compelling evidence linking ET1 to obesity-related vascular pathology, key questions remain regarding its exact role in cardiometabolic disease. Plasma ET1 levels are elevated in obese individuals compared to their lean counterparts, including both adults and adolescents [18,19]. However, it remains unclear whether these increased levels result from heightened ET1 production or impaired clearance, as ET1 is predominantly secreted into the interstitial space within tissues [20,21]. Moreover, the cellular origin of ET1 within adipose tissue remains debated, with conflicting evidence regarding whether it primarily derives from adipocytes or vascular endothelial cells. Interestingly, both ET1 and TSP1 are modulated by hypoxia [22], reinforcing the potential mechanistic link between these two proteins. However, further research is needed to elucidate their precise roles in obesity-related cardiometabolic dysfunction, particularly in women.

Sex differences in fat distribution may further influence these metabolic and vascular interactions. Women of the same age and BMI as men tend to accumulate more subcutaneous adipose tissue, particularly in the lower body. In contrast, men exhibit greater visceral adiposity, characterized by increased abdominal fat deposition [23]. This sex-specific distribution is largely governed by estrogens, which exert cardioprotective effects by reducing inflammation and modulating sympathetic nervous system activity. However, following menopause, estrogen levels decline, leading to a shift in fat distribution that resembles the male pattern and increasing cardiovascular risk. While estrogens’ protective effects are well-documented, they appear to be diminished in obese women of reproductive age [24]. Nevertheless, epidemiological evidence suggests that, prior to menopause, women have a lower risk of obesity-related cardiovascular disease compared to men. Notably, the onset of atherosclerotic vascular lesions is delayed by approximately 10 years in women [25]. Contrary to the assumption that excess adipose tissue enhances estrogen production via increased aromatase activity, studies have found an inverse relationship between BMI and estradiol levels in premenopausal women. Additionally, adipose-derived factors involved in inflammation, vascular function, and immune regulation have been implicated in sex-specific cardiovascular risk [26].

## 2. Materials and Methods

### 2.1. Study Population

This cross-sectional observational study included 55 adult Caucasian premenopausal women with obesity who were consecutively recruited from the Endocrinology Unit of “R. Dulbecco” University Hospital, Catanzaro, Italy, while undergoing an oral glucose tolerance test (OGTT) to define their glycometabolic status. At the time of recruitment, clinical data were collected for all patients, including their family history of diabetes mellitus, smoking habits, and hypertension, and B-mode ultrasound screening was performed to detect mild/severe hepatic steatosis, in consideration of the high diagnostic accuracy of this technique [27]. Eligible participants were 18 to 55 years of age with a body mass index (BMI) ≥30 kg/m^2^. Patients were excluded if they met any of the following conditions: a diagnosis of diabetes mellitus; current pregnancy; a history of cardiovascular events or very high cardiovascular risk (i.e., carotid stenosis, atrial fibrillation, heart failure, or peripheral arterial disease); renal insufficiency; uncontrolled hypothyroidism or hyperthyroidism; secondary causes of obesity, including Cushing’s syndrome or lipodystrophy; acute inflammation (i.e., leukocytosis >10,000/mm^3^, erythrocyte sedimentation rate [ESR] >30 mm/h); connective tissue diseases; a history of malignancies (except for non-melanoma skin cancers or low-risk thyroid cancers in complete remission for at least three years); or the use of estrogen-progestin therapy; chronic treatment with steroids, metformin, or antiobesity medications (i.e., GLP-1 Receptor Agonists) within the last three months [28]. However, the use of hypolipidemic or antihypertensive agents was not an exclusion criterion, and the relevant medication data were collected. Participants were categorized into two groups based on the WHO classification for individuals of European ancestry, which defines obesity-related disease risk by BMI [29]. Women with a BMI of 30–34.9 kg/m^2^ (Class I obesity) were classified as having low-risk obesity, and those with a BMI ≥ 35 kg/m^2^ (Class II and III obesity) were grouped as moderate/high-risk obesity.

### 2.2. Laboratory Assessments

Anthropometric parameters (weight, height, and waist circumference) and blood pressure were recorded, and venous blood samples were obtained from fasting participants during the OGTT. Routine hematological, coagulative, and biochemical tests—including hematocrit (Ht), hemoglobin (Hb), red blood cell count (RBC), white blood cell count (WBC), platelet count (PLT), prothrombin time (PT), activated partial thromboplastin time (aPTT), fibrinogen, D-dimer, fasting glucose, insulin, glycated hemoglobin (HbA1c), liver function tests (AST, ALT, GammaGT), total and HDL cholesterol, triglycerides, and ESR—were performed immediately. Serum aliquots were collected post-centrifugation and stored at −80 °C for subsequent measurements of TSP1 and ET1. HbA1c levels were quantified using boronate affinity chromatography (Hb9210, Menarini Diagnostics, Florence, Italy), and insulin concentrations were determined by immunochemiluminescence (ADVIA Centaur XP, Siemens, Berlin, Germany). Glucose, AST, ALT, GammaGT, creatinine, ESR, and lipid profiles were analyzed using a Cobas 8000 system (Roche Diagnostics, Rotkreuz, Switzerland). Blood counts were determined by ADVIA 2120i (Siemens Healthcare Diagnostics, Berlin, Germany). Hemostatic parameters were evaluated by BCS XP (Siemens Healthcare Diagnostics, Berlin, Germany) according to the manufacturer’s protocol. TSP1 and ET1 levels were measured on the Ella™ automated ELISA platform (ProteinSimple, Bio-Techne, San Jose, CA, USA) for protein quantification, according to the manufacturer’s protocol. Ella™system performs immunoassays using a microfluidic Simple Plex cartridge pre-loaded with a capture monoclonal antibody specific for human TSP1 and ET1 (R&D Systems, Minneapolis, MN, USA). Serum samples (10 µL for TSP1 and 50 µL for ET1, respectively) were run through a microfluidic channel that binds the protein of interest and measures each analyte in triplicates (intra-assay CV: <3% and <4% and inter-assay CV: <8% and <10% for ET1 and TSP1, respectively). Relative values for each protein were obtained on a predetermined standard curve. Sensitivity for TSP1 was 91.7 pg/mL (assay range: 498–303, 880 pg/mL), and sensitivity for ET1 was 0.11 pg/mL (assay range: 0.26–1000 pg/mL).

### 2.3. Statistical Analysis

Data were presented as the median and interquartile range (IQR) and analyzed with the non-parametric Mann–Whitney U test. Categorical variables were presented as counts and percentages, with differences assessed via chi-square or Fisher’s exact test, as appropriate. Correlation analyses between continuous variables, including TSP1, ET1, and metabolic/inflammatory parameters, were performed using Spearman’s rank correlation (r). A *p*-value of <0.05 was considered statistically significant for all tests. To evaluate potential confounding by platelet count and functions in TSP1 measurements, a PLT-corrected index was calculated as the ratio of TSP1 levels (pg/mL) to PLT (*n* × 10^3^) and compared across BMI subgroups (30–34.9 vs. ≥35 kg/m^2^). All analyses were conducted using JASP Graphical Statistical Software Version 0.17.1.0 (University of Amsterdam, Amsterdam, The Netherlands) based on R Stats packages. Multiple testing corrections were not performed, as the analyses were intended to be exploratory in consideration of the pilot design of the study.

## 3. Results

### 3.1. Clinical Characteristics of the Study Population

Fifty-five nondiabetic women with obesity were recruited at the Endocrinology Unit to identify novel biomarkers of insulin resistance and associated metabolic complications. The median age of the cohort was 39 years (IQR, 25.0–47.5). The median body weight was 89 kg (IQR, 81.5–103.7), the median height was 1.63 m (IQR, 1.58–1.68), and the median BMI was 33.7 kg/m^2^ (IQR, 30.8–38.6). Notably, 52% of participants had a BMI of ≥35 kg/m^2^, indicating a high prevalence of moderate/high obesity. The median waist circumference was 99.5 cm (IQR, 94.5–107.3), indicative of marked visceral adiposity. Systolic blood pressure (SBP) was measured at a median of 110 mmHg (IQR, 110–120) and diastolic blood pressure (DBP) 75 mmHg (range, 70–80). Overall, 12% of participants met the criteria for hypertension and were under treatment with renin angiotensin system-acting agents and/or beta-blockers. A family history of diabetes was documented in 58.8% of cases, while 16.4% presented impaired glucose regulation (IFG or IGT). Hepatic steatosis was detected in 40.9% of the cohort, and 18.1% of participants were active or former smokers (whereas 81.9% were non-smokers) (Table 1).

### 3.2. Metabolic and Inflammatory Indices, Hepatic and Lipid Parameters

The median fasting blood glucose was 88.0 mg/dL (IQR, 84.0–93.0), and the 2 h post-OGTT glucose measured 113.5 mg/dL (IQR, 96.8–127.3), indicating no overt diabetes. However, fasting insulin levels were elevated (median, 14.0 mU/L; IQR, 10.0–20.0), corresponding to a median HOMA-IR of 3.22 (IQR, 2.05–4.44). A substantial proportion (69.8%) had HOMA-IR values above 2.5, consistent with a widespread IR state, confirmed by hyperinsulinemic responses during the OGTT. The median HbA1c was 5.50% (IQR, 5.24–5.60), remaining within nondiabetic ranges (Table 1). Median AST and ALT levels were each 20.0 U/L (AST IQR, 16.0–25.0; ALT IQR, 14.0–29.0), while GammaGT measured a median of 17.0 U/L (IQR, 13.0–22.0). The median total cholesterol was 176.0 mg/dL (IQR, 160.0–192.0), median LDL 113.0 mg/dL (IQR, 96.0–130.0), median HDL 53.0 mg/dL (IQR, 43.0–59.0), and median triglycerides 83.0 mg/dL (IQR, 63.8–123.8). Although these values were largely within normal levels, some participants exhibited low HDL, consistent with obesity-related metabolic syndrome (Table 1), and two of them were under lipid-lowering medications.

A moderate inflammatory and pro-coagulative state, typical of metabolic disorders in obesity, was suggested by a median ESR of 16 mm/h (IQR, 9–25) and a median fibrinogen concentration of 361.5 mg/dL (IQR, 328.3–389.8). The median D-dimer was 0.30 mg/L (IQR, 0.24–0.37).

### 3.3. Thrombospondin-1 (TSP1) and Endothelin-1 (ET1)

**Figure 1:**
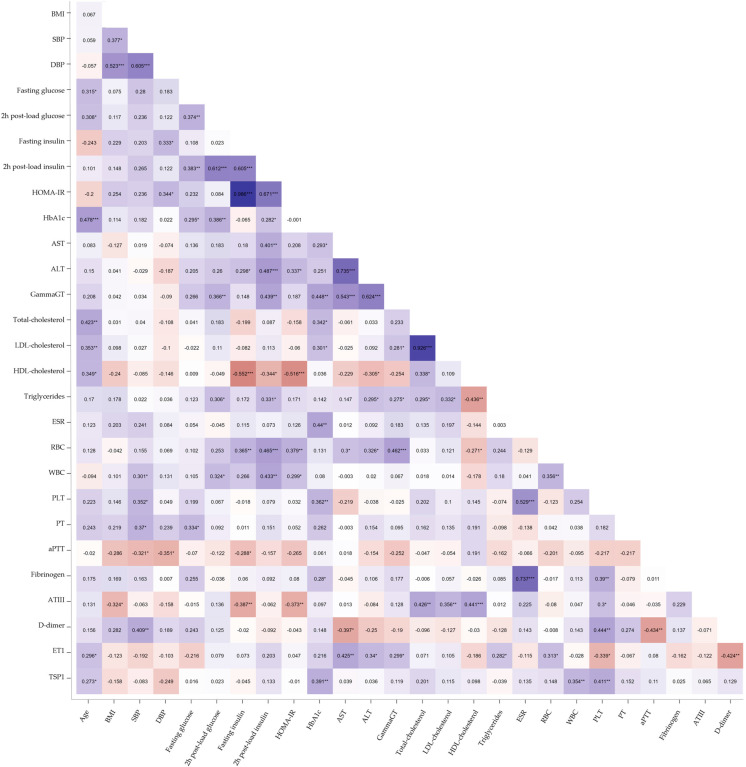
Heatmap of Spearman’s correlation analysis between ET1, TSP1, and clinical, metabolic, and hemato-coagulative parameters in obese women. The blue shades represent negative correlations, the red shades indicate positive correlations, and the white denotes no correlation. The intensity of the color reflects the absolute magnitude of the correlation coefficient (r). Statistically significant correlations are marked with asterisks: * *p* < 0.05; ** *p* < 0.01; *** *p* < 0.001.

Two emerging markers of cardiometabolic risk—ET1 (median, 2.050 pg/mL; IQR, 1.695–2.565) and TSP1 (median, 2.967 × 10^7^ pg/mL; IQR, 2.159 × 10^7^–4.045 × 10^7^)—were evaluated by automated ELISA. The Spearman correlation revealed that ET1 was positively correlated with AST (r = 0.425, *p* < 0.01), ALT (r = 0.340, *p* < 0.05), Gamma-GT (r = 0.299, *p* < 0.05), and triglycerides (r = 0.282, *p* < 0.05) in premenopausal women with obesity. Conversely, ET1 demonstrated significant negative correlations with D-dimer (r = −0.424, *p* < 0.01) and platelet count (PLT, r = −0.339, *p* < 0.05) (Figure 1). TSP1 correlated positively with age (r = 0.273, *p* < 0.05), HbA1c (r = 0.391, *p* < 0.01), and PLT (r = 0.411, *p* < 0.01). A positive correlation with white blood cell count (WBC, r = 0.354, *p* < 0.01) also emerged, suggesting a potential role in obesity-related inflammation (Figure 1). Given the strong correlation between TSP1 and PLT, an index incorporating platelet count was developed to better delineate the potential independent effects of TSP1 released by dysfunctional adipose tissue. Changes in PLT count and function have been widely reported in chronic inflammatory diseases and immunological disorders, and simple ratios combining nutritional status markers with PLT count have shown high diagnostic accuracy for assessing disease activity [30,31,32]. Based on this evidence, we investigated the TSP1-to-PLT ratio to explore its variations during obesity progression and its potential relevance as a biomarker of cardiometabolic risk.

### 3.4. Comparison by Obesity Severity

**Figure 2:**
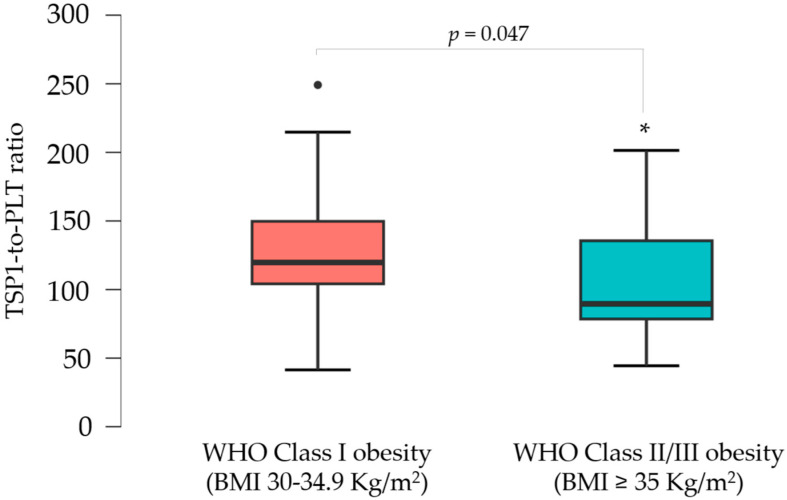
Boxplots illustrating the distribution of the TSP1-to-PLT1 ratios in women with WHO Class I vs. Class II/III obesity. * *p* < 0.05.

A subgroup analysis was performed based on BMI classification. Women with WHO Class II/III obesity (BMI ≥ 35 kg/m^2^) showed significantly higher median body weight (107.0 kg vs. 82.5 kg, *p* < 0.001), BMI (39.1 vs. 31.0, *p* < 0.001), and waist circumference (107.0 cm vs. 96.5 cm, *p* < 0.001) with respect to women with WHO Class I obesity (BMI 30–34.9 kg/m^2^). They also had higher SBP (120 mmHg vs. 110 mmHg, *p* = 0.010) and DBP (80 mmHg vs. 70 mmHg, *p* < 0.001). Furthermore, the median levels of ESR (18 mm/h vs. 11 mm/h, *p* = 0.041) were significantly higher among those women with WHO Class II/III obesity, along with a tendency towards higher HOMA-IR values (3.28 vs. 2.73, *p* = 0.097) and lower TSP1 and ET1 levels, suggesting the potential effect of obesity severity on these parameters, even before significant alterations in glucose levels, lipid profiles, liver enzymes, or coagulation parameters were observed during disease progression (Table 2). To account for possible intergroup differences in platelet counts and activation, the TSP1-to-PLT ratio was evaluated. This PLT-corrected index for TSP1 was significantly lower in the moderate/high-risk obesity subgroup (89.56 vs. 119.73, *p* = 0.047) (Figure 2). 

Overall, women of reproductive age with severe increments in BMI (WHO Class II/III obesity) exhibited more pronounced metabolic dysfunction and inflammatory activation compared to those with milder excess body weight (WHO Class I obesity). The variation in TSP1 levels with obesity progression suggests a potential pathogenic function for this glycoprotein in the cardiometabolic sequelae of obesity and insulin resistance before the menopausal transition.

## 4. Discussion

The findings herein underscore TSP1 as a potentially important biomarker of metabolic and inflammatory complications in obesity, particularly in premenopausal women. The positive correlation of TSP1 with both inflammatory (WBC) and metabolic (HbA1c) parameters points to a link between TSP1 and the chronic inflammatory milieu characteristic of obesity. The strong association between TSP1 and platelet count motivated the development of a PLT-corrected index, which showed significant differences in women with severe obesity. Prior evidence supports the role of TSP1 beyond hemostasis regulation, indicating its involvement in inflammation, insulin resistance, and angiogenesis [5,14]. For instance, TSP1 has been implicated in promoting inflammation through TLR4 activation, driving the release of pro-inflammatory cytokines such as TNF-α [10]. In murine models, TSP1 deficiency has been shown to reduce inflammation and enhance insulin sensitivity, suggesting its potential as a therapeutic target [7].

In humans, elevated TSP1 levels have been associated with visceral obesity, hypertension, and metabolic disturbances, emphasizing its central role in maintaining inflammatory responses and metabolic dysfunction [2,5]. Nonetheless, the relationship between TSP1 expression and obesity severity remains somewhat complex. In this study, which is the first of its kind to stratify an obese cohort of women by BMI, those with WHO Class II/III obesity manifested poorer metabolic and inflammatory profiles. These women demonstrated higher ESR and a tendency towards higher HOMA-IR and D-dimer levels, indicative of increased cardiometabolic risk and systemic inflammation. The reduced TSP1-to-PLT ratio may reflect heightened consumption or dysregulated production of TSP1 in the context of severe obesity. Former in vitro studies conducted by our group support this hypothesis, demonstrating that hypoxia—a hallmark of visceral adipose tissue—downregulates TSP1 expression in murine adipocytes [8]. This effect is likely exacerbated by increased BMI and visceral fat accumulation in human obesity, as the progression from WHO Class I to WHO Class II and III obesity is associated with more hypoxic, dysfunctional adipose tissue that is increasingly resistant to insulin, regardless of patient sex [33]. Our research further reveals that the protein secretion profile of murine adipocytes under hypoxic conditions mirrors circulating protein patterns in humans. For instance, prothymosin-alpha, a pro-inflammatory factor upregulated by approximately 50% under hypoxia, is also increased by a similar margin in the serum of obese, nondiabetic adult women with metabolic profiles comparable to the participants in the current study [34]. These findings suggest that hypoxia in visceral adipose tissue may drive the altered secretion of proteins, including TSP1, in obesity. Although normal-weight individuals were not included as controls in this study, preliminary data from our lab suggest a trend toward lower TSP1 levels in age-matched, normal-weight patients. This aligns with their lower baseline cardiovascular risk and the relatively normoxic state of their adipose tissue compared to individuals with obesity.

ET1, another emerging marker evaluated in this study, was positively correlated with hepatic enzymes and triglycerides, suggesting a possible role in obesity-related hepatic and lipid alterations. However, no significant difference in ET1 levels was observed when comparing the subgroup of women with ultrasound-confirmed hepatic steatosis to those without. The negative correlation of ET1 with D-dimer and platelet count may reflect a complex interplay between endothelial function and coagulation pathways. These findings align partially with a prior study that focused exclusively on male subjects [18]. In that study, obese normotensive men exhibited slightly higher circulating ET1 levels compared to non-obese, normotensive men, although no correlation between ET1 levels and BMI or fasting glucose was observed. Notably, differences between hypertensive and normotensive obese male patients were also observed [18]. Preliminary analyses of the hypertensive subset in our cohort of premenopausal women similarly suggest higher median ET1 levels compared to normotensive participants. Interestingly, no correlation between TSP1 and ET1 was found in our study. The systemic vasoconstrictor effect of ET1 is influenced by TSP1, which facilitates ET1 binding to its receptor, suggesting that elevated TSP1 levels may contribute to increased vascular tone and, consequently, higher thrombotic and cardiovascular risk [2]. At present, we cannot exclude that the observed downregulation of TSP1 and ET1 in this cohort of premenopausal women may represent a transient compensatory mechanism to mitigate cardiovascular risk in the context of WHO Class II/III obesity [34].

Given the proposed roles of TSP1 in insulin resistance and low-grade chronic inflammation [5,6], this molecule may represent a promising target for both pharmacological and lifestyle interventions (i.e., exercise and caloric restriction). Furthermore, quantification of TSP1 and ET1 could assist in identifying obese patients at higher risk of cardiometabolic complications. Our data, although preliminary, suggest age- and comorbidity-dependent variability in the potential pathogenic role of TSP1, highlighting the need for further investigation to refine personalized strategies for obesity prevention and treatment.

We recognize the small sample size of the cohort as a limitation of this study, which was of an exploratory nature. Validation of the results in a larger, more diverse population, including both male and female participants, is currently in progress. Furthermore, the cross-sectional design limits the ability to establish causal relationships in the metabolic alterations associated with obesity, whereas the lack of data on supplements and micronutrients that may influence TSP1 expression prevents adjustment for these potential confounders. Similarly, the absence of data on platelet activation and subpopulations may limit adjustments for obesity-related shifts in platelet function, except for platelet count. Finally, we measured total TSP1 levels without assessing its functional state, including CD47 receptor binding and biological activity, which warrants further investigation.

In conclusion, our results hint at a pivotal role for TSP1 and ET1 in the complex interplay between obesity, inflammation, and insulin resistance in women. Beyond their potential as biomarkers for obesity-related disease risk, such insights hold promise for the development of innovative preventive and therapeutic measures against the cardiometabolic sequelae of obesity.

## Figures and Tables

**Table 1:** Clinical and laboratory characteristics of the study population.

Total Study Population Characteristics	Median	IQR	*n* (%)
Age, years	39.0	25.0–47.5	
Family history of diabetes, *n*			30 (58.8)
Smoking status			
Current smoker, n			9 (17.6)
Ex-smoker, n			1 (2.0)
Weight, Kg	89.0	81.5–103.7	
Height, m	1.63	1.58–1.68	
BMI, Kg/m^2^	33.7	30.8–38.6	
Waist circumference, cm	99.5	94.5–107.3	
Hypertension, *n*			6 (12.0)
SBP, mmHg	110	110–120	
DBP, mmHg	75	70–80	
Hepatic steatosis, *n*			18 (40.9)
Fasting glucose, mg/dL	88.0	84.0–93.0	
2 h post-load glucose, mg/dL	113.5	96.8–127.3	
Fasting insulin, mU/L	14.0	10.0–20.0	
2 h post-load insulin, mU/L	90.0	56.0–133.0	
IFG and/or IGT, *n*			9 (16.4)
HOMA-IR	3.22	2.05–4.44	
HOMA-IR > 2.5, *n*			37 (69.8)
HbA1c, %	5.50	5.24–5.60	
AST, U/L	20.0	16.0–25.0	
ALT, U/L	20.0	14.0–29.0	
GammaGT, U/L	17.0	13.0–22.0	
Total-cholesterol, mg/dL	176.0	160.0–192.0	
LDL-cholesterol, mg/dL	113.0	96.0–130.0	
HDL-cholesterol, mg/dL	53.0	43.0–59.0	
Triglycerides, mg/dL	83.0	63.0–123.0	
ESR, mm	16	9–25	
HT, %	39.8	37.8–42.4	
Hb, g/dL	13.3	12.5–14.1	
RBC, *n* × 10^3^	4.7	4.5–5.1	
WBC, *n* × 10^3^	6.9	5.9–7.5	
PLT, *n* × 10^3^	267.5	223.5–316.8	
PT, s	104.0	99.0–112.0	
aPTT, s	29.0	27.0–30.0	
Fibrinogen, mg/dL	361.5	328.3–389.8	
ATIII, %	98.0	93.0–105.8	
D-dimer, mg/L	0.30	0.24–0.37	
ET1, pg/mL	2.050	1.695–2.565	
TSP1, pg/mL	2.967 × 10^7^	2.159 × 10^7^–4.045 × 10^7^	

Data are presented as the median and interquartile range (IQR) or as a number (*n*) and percentage (%), as appropriate. BMI, body mass index; SBP, systolic blood pressure; DBP, diastolic blood pressure; IFG, impaired fasting glucose; IGT, impaired glucose tolerance; HOMA-IR, homeostatic model assessment of insulin resistance; HbA1c, glycated hemoglobin; AST, aspartate aminotransferase; ALT, alanine aminotransferase; GammaGT, gamma glutamyltransferase; ESR, erythrocyte sedimentation rate; HT, hematocrit; Hb, hemoglobin; RBC, red blood cell count; WBC, white blood cell count; PLT, platelet count; PT, prothrombin time; aPTT, activated partial thromboplastin time; ATIII, antithrombin III; ET1, endothelin-1; TSP1, thrombospondin-1.

**Table 2:** Comparison of clinical and laboratory characteristics of the study population by obesity severity.

Group Characteristics	WHO Class I Obesity			WHO Class II/III Obesity			*p* Value
	Median	IQR	N (%)	Median	IQR	N (%)	
Age, years	29.5	24.3–47.5		40.0	28.0–45.0		0.716
Family history of diabetes, *n*			15 (57.7)			15 (51.7)	0.657
Smoking status							
Current smoker, *n*			5 (19.3)			4 (13.7)	0.848
Ex-smoker, *n*			0 (0.0)			1 (4.8)	
Weight, Kg	82.5	77.9–84.9		107.0	96.0–111.0		**<0.001**
Height, m	1.63	1.59–1.65		1.60	1.58–1.69		0.923
BMI, Kg/m^2^	31.0	30.0–32.5		39.1	37.0–42.1		**<0.001**
Waist circumference, cm	96.5	92.0–98.0		107.0	100.8–113.8		**<0.001**
Hypertension, *n*			3 (11.5)			3 (10.3)	1.000
SBP, mmHg	110	110–120		120	110–126		**0.010**
DBP, mmHg	70	65–75		80	76–83		**<0.001**
TSH, mU/L	2.14	1.18–3.04		2.69	1.40–3.35		0.578
Hepatic steatosis, *n*			8 (30.8)			10 (34.4)	0.769
Fasting glucose, mg/dL	86.5	84.0–93.0		88.0	86.0–93.0		0.548
2 h post-load glucose, mg/dl	101.5	95.3–122.3		118.5	101.5–130.8		0.195
Fasting insulin, mU/L	12.5	9.0–18.8		15.0	12.0–21.0		0.113
2 h post-load insulin, mU/L	79.0	37.0–97.0		74.0	57.0–133.0		0.275
IFG and/or IGT, *n*			3 (11.5)			6 (20.7)	0.839
HOMA-IR	2.73	1.84–3.91		3.28	2.67–4.67		0.097
HOMA-IR > 2.5, *n*			15 (57.7)			22 (75.8)	0.151
HbA1c, %	5.40	5.20–5.60		5.60	5.20–5.70		0.365
AST, U/L	20.0	16.0–25.0		19.0	15.0–24.0		0.691
ALT, U/L	18.5	14.0–30.8		19.0	16.0–28.0		0.872
GammaGT, U/L	15.0	13.3–22.0		18.0	13.0–20.0		0.864
Total-cholesterol, mg/dL	167.5	157.0–197.8		178.0	159.0–185.0		0.991
LDL-cholesterol, mg/dL	108.0	94.3–124.8		118.0	96.0–124.0		0.692
HDL-cholesterol, mg/dL	55.0	44.0–65.5		49.0	46.0–55.0		0.118
Triglycerides, mg/dL	74.5	59.3–111.0		90.0	67.0–120.0		0.304
ESR, mm	11	8–19		18	13–25		**0.032**
HT, %	41.0	38.2–42.4		39.1	37.7–42.0		0.487
Hb, g/dL	13.6	12.6–14.1		13.3	12.6–13.4		0.507
RBC, *n* × 10^3^	4.8	4.6–5.1		4.7	4.4–4.9		0.593
WBC, *n* × 10^3^	6.7	5.1–7.5		7.0	6.3–7.7		0.246
PLT, *n* × 10^3^	241.5	196.8–298.5		258.0	230.0–298.0		0.275
PT, s	103.5	94.3–113.0		106.0	102.0–112.0		0.284
aPTT, s	29.0	27.3–31.0		28.0	26.0–29.0		0.078
Fibrinogen, mg/dL	337.5	314.8–382.8		363.0	330.0–378.0		0.121
ATIII, %	100.0	93.3–105.8		95.0	90.0–99.0		0.170
D-dimer, mg/L	0.28	0.23–0.33		0.33	0.29–0.44		0.092
ET1, pg/mL	2.060	1.722–2.587		1.800	1.540–2.280		0.386
TSP1, pg/mL	3.057 × 10^7^	2.172 × 10^7^–4.104 × 10^7^		2.454 × 10^7^	1.837 × 10^7^–3.567 × 10^7^		0.197

Data are presented as the median and interquartile range (IQR) or as a number (N) and percentage (%). Intergroup comparisons were conducted using the Mann–Whitney U test, the Chi-square test, or Fisher’s exact test, as appropriate. Bold values denote statistical significance at *p* level < 0.05.

## Data Availability

Data supporting the study findings are available to the corresponding authors upon request.
